# Towards a Practical Implementation of a Single-Beam All-Optical Non-Zero-Field Magnetic Sensor for Magnetoencephalographic Complexes

**DOI:** 10.3390/s22249862

**Published:** 2022-12-15

**Authors:** Mikhail Petrenko, Anton Vershovskii

**Affiliations:** Ioffe Institute, Russian Academy of Sciences, 194021 St. Petersburg, Russia

**Keywords:** optically detected magnetic resonance, quantum magnetometer, magnetoencephalography

## Abstract

We present a single-beam all-optical two-channel magnetic sensor scheme developed for biological applications such as non-zero-field magnetoencephalography and magnetocardiography. The pumping, excitation and detection of magnetic resonance in two cells are performed using a single laser beam with time-modulated linear polarization: the linear polarization of the beam switches to orthogonal every half-cycle of the Larmor frequency. Light with such characteristics can be transmitted over a single-mode polarization-maintaining fiber without any loss in the quality of the polarization characteristics. We also present an algorithm for calculating optical elements in a sensor scheme, the results of measuring the parametric dependences of magnetic resonance in cells, and the results of direct testing of a sensor in a magnetic shield. We demonstrate sensitivity at the level of 20 fT/√Hz in one sensor channel in the frequency range of 80–200 Hz.

## 1. Introduction

One of the most notable challenges of our time is the task of investigating ultra-weak magnetic fields of the brain. The set of scientific methods that provide a solution to this problem is called magnetoencephalography (MEG) [1,2]. The avalanche growth of interest in this problem, which has manifested itself over the past ten years, is mainly associated with the advent of compact, optical magnetic field sensors. The principle of operation of these sensors is based on the effect of magnetic resonance (MR) [3,4,5]. The application of these sensors to MEG problems has shaken the long-term monopoly of superconducting SQUID (superconducting quantum interference device) systems [6,7] and made it possible to overcome their inherent limitations.

The first (and still the most sensitive) optical sensors capable of competing with SQUID systems were sensors based on the SERF (spin exchange relaxation-free) effect [8,9,10,11,12,13,14,15]. These are zero-field since they operate only in a zero magnetic field, that is, in stationary magnetically shielded rooms. After SERF sensors convincingly demonstrated their competitiveness in MEG tasks, a number of research groups began to explore the possibility of adapting non-zero-field sensors to MEG tasks. These sensors are initially characterized by somewhat less sensitivity than SERF sensors. Still, their use would make it possible to drastically reduce the requirements for suppressing the external field and its spatial gradients. This, in turn, would make it possible to replace expensive magnetically shielded rooms with magnetic shields and, in the future, to do without shields at all [16,17,18,19,20,21,22]. The possibilities and prospects for the use of scalar non-zero-field optical magnetometers (the class to which the sensor presented in this work belongs) were studied in [20] and partially in [23]. A recent review [24] summarizes the general aspects of optical and magnetic field sensors and the problems associated with applications to biomagnetic measurements.

This paper presents a scheme of such a sensor, a single-beam all-optical non-zero-field two-channel magnetometer, i.e., a magnetometer-gradientometer of a non-zero field. The sensor is built in accordance with the principles we outlined earlier in [25,26]; it meets the MEG requirements for all the main parameters, namely, for sensitivity, speed and ability to function without creating RF interference to adjacent sensors.

## 2. Materials and Methods

The scheme proposed by us in [25] is extremely simple and compact. This advantage is due to several factors.

First, it uses a single beam with modulated (from partial left circular polarization to linear and then to partial right circular polarization) ellipticity for pumping, excitation, and detection of the MR. This scheme differs from numerous single-beam schemes proposed earlier [27,28,29] by the absence of sensitivity-reducing compromises. Pumping by the circularly polarized component and detection by the linearly polarized radiation component are separated in time, according to the phases of the Larmor precession. The ellipticity of the output beam changes its sign during the modulation period and acquires the maximum absolute value twice during the period *T_M_* = 2π/ω*_M_* [25]. The optimal values of maximum ellipticity lie in the range of 15–20°, meaning that the linear component is always present in the beam. Twice per period, the polarization becomes purely linear (π), with the polarization azimuth corresponding to the polarization azimuth of the incoming beam. For the purposes of the following discussion, radiation can be considered as the sum of two components, purely linear (π) and purely circular (σ^±^), characterized by time-modulated intensities. This type of modulation is achieved using an electro-optical modulator (EOM). This allows for pumping and detection to be carried out with the highest possible efficiency.

Second, we use combined (hyperfine + Zeeman) pumping, first proposed in [30] and theoretically justified in [31]. The frequency of the beam is tuned to the D_1_ optical line of the alkali metal line; it links the hyperfine level *F = I* − ½ of the ground state *S*_1/2_ of the atom with levels *F’ = I* ± ½ of the nearest excited state *P*_1/2_ [30,31]. The effective Zeeman pumping of the *F = I* + ½, *m_F_ = F* sublevel is due to the partial conservation of momentum in the excited state: the electronic part of the momentum is completely destroyed in collisions with the buffer gas, but the nuclear component is predominantly preserved [31].

Third, we use a modification of the *M_x_* design, known as the Bell–Bloom scheme [4,32]. In this modification, the excitation of the MR is carried out by modulating the circular component of the pumping light at the Larmor frequency. This makes it possible to perform the excitation without a resonant radio-frequency field and, as a result, eliminate the interference such a field creates.

Fourth, we use strong optical pumping, which allows us to collect most of the atoms at the level *F* = *I* + ½, *m_F_* = *F*. William Happer called this state “end-state” or “stretched”, and showed [33] that the spin-exchange rate in this state can decrease significantly. Indeed, as the pump intensity increases, the broadening of the magnetic resonance is preceded by its narrowing [34], which makes it possible to bring the sensitivity of the nonzero field sensor closer to that of the SERF sensor to some extent.

Finally, we detect MR at the transition *F = I* + 1/2, *m_F_ = F* ↔ *F* – 1 of the ground state by rotating the polarization angle of the linearly polarized (π) radiation component [35,36]. Therefore, the π-component of the beam is detuned in frequency from the interrogated optical transition by the hyperfine splitting of the ground state (for Cs, this is 9.192 GHz). Thus, the conditions for quantum non-demolition measurement (QND) are realized.

Thus, we simultaneously achieve near-optimal conditions for both optical pumping and MR excitation and detection. However, adapting the scheme [25] for application in MEG sensors is associated with certain difficulties. Since light contains both linearly and circularly polarized components, it cannot be transmitted through an optical fiber [37] without deteriorating its polarization characteristics. The obvious solution is to use a separate EOM in each sensor, which can significantly increase the cost of a multichannel MEG complex. On the contrary, the use of a common (sufficiently powerful compared to VCSEL lasers used in SERF zero-field sensors) pump source with a common EOM for several sensors would not only significantly simplify and reduce the cost of the MEG complex but also reduce technical noise by suppressing the common light noise.

In [38], we proposed a modification of the scheme, which will subsequently allow using a standard single-mode polarization-maintaining (SM-PM) optical fiber to solve this problem. Such a fiber has two eigenmodes characterized by orthogonal (*s* and *p*) polarizations propagating along the fiber’s axis [39]. The phase delay between the modes is not fixed and can change when the fiber is bent, preventing radiation transmission with elliptical polarization. Nothing, however, prevents the transmission of *linearly polarized radiation with modulated azimuth* over the SM-PM fiber. The azimuth of the polarization is modulated as follows: *s*-polarization is transmitted through the fiber during the first half-cycle of the Larmor frequency, while *p*-polarization is transmitted during the second half-cycle (note that we do not impose any requirements on the stability of the phase delay between these two half-cycles). Now the problem is reduced to ensuring that this radiation can be converted into radiation containing π and σ ± components, properly modulated in intensity. As will be shown below, such a conversion can be achieved using a combination of a quarter-wave plate (QWP) and a regulated linear polarizer.

**Figure 1:**
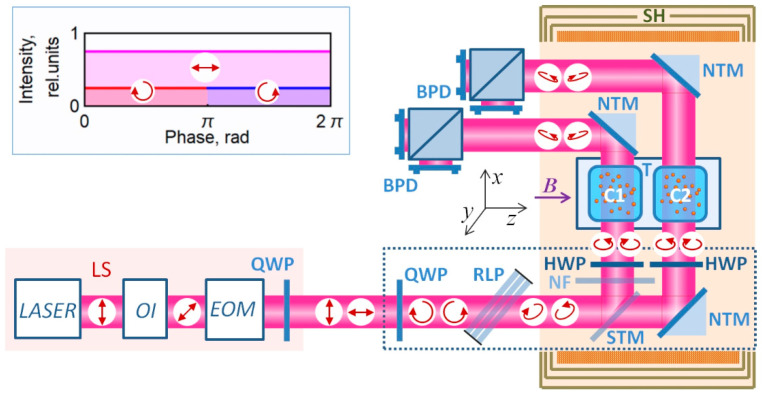
Simplified scheme of the experiment: LS—radiation source, OI—optical isolator, EOM—electro-optical polarization modulator, QWP—quarter-wave plate, RLP—regulated linear polarizer, NF—neutral filter, HWP—half-wave plates, C1, C2—gas cells with Cs vapors, STM is a semitransparent mirror, NTM is a non-transparent (opaque) mirror, BPD are balanced photodetectors, T is a thermostat, SH is a magnetic shield with a solenoid. Arrows indicate beam polarization states corresponding to two modulation half-cycles. Inset: time diagram of the polarization composition of the beam during one modulation period.

This paper presents a scheme of a single-beam all-optical non-zero field two-channel magnetometer-gradientometer (Figure 1) with two channels pumped and interrogated by one common beam; we also present a general algorithm for calculating the optical scheme of the sensor and the results of a study of its characteristics.

The measurements were carried out on the setup described in [23,25,40] and modified in accordance with the task of the experiment. The light source (LS) consisted of an external cavity diode laser (VitaWave ECDL 895R) generating about 25 mW at a wavelength of 894.592 nm, an optical isolator, and an electro-optical modulator (Thorlabs EO-AM-NR-C1). The control voltage at the EOM, modulated at a frequency of ~42 kHz with an amplitude of 200 V, provided a phase shift of ±45° between the components of the light decomposed along the EOM’s own axes. An additional QWP (quarter-wave plate) provided linearly polarized radiation with modulated azimuth generation at the output of the radiation source.

The sensitive elements of the gradiometric sensor were cubic cells 8 × 8 × 8 mm^3^ in size, containing saturated cesium vapor and nitrogen at a pressure of ~100 torr. A thermostat with cells and a heater was placed in the central region of a multilayer magnetic shield. A magnetic field induction of ~12 μT was maintained in the shield. A quarter-wave plate (QWP) installed at the sensor input converts linearly polarized radiation with modulated azimuth into radiation with switchable (from left to right and vice versa) circular polarization (the angle between the QWP axes and the fiber’s own axes is 45°). Further, the regulated linear polarizer converts the circular polarization into an elliptical one, and the linear component necessary for detection appears in the beam. The linear polarizer used in our experiment is a stack of plane-parallel glass plates fixed at a Brewster angle to the beam direction in a common frame. The polarizer is adjusted by changing the number of plates. The angle of rotation of the frame around the beam determines the polarization azimuth of the π component. Unfortunately, in our experiment, the power of the laser source (taking into account the losses introduced by additional optical elements) turned out to be insufficient to ensure the optimal light intensity for pumping and interrogating two channels of the gradiometer. This prevented us from using SM-PM fiber. Instead, we had to confine ourselves to a model experiment, i.e., to reproduce at the output of the light source those characteristics that can certainly be obtained at the output of an ideal SM-PM fiber.

Half-wave plates (HWP) are installed in such a way as to ensure the optimal azimuth of the π-component of radiation in the cells with respect to the direction of the magnetic field vector. In our experiment, the linear polarizer was positioned in such a way that the electric vector ***E*** of the linear radiation component was parallel to the field vector ***B***. When D_1_ line is used for the pump, the above makes it possible to minimize the broadening of the MR by the linear radiation component by eliminating its destructive interaction with the most populated (as a result of optical pumping) levels *F = I* + 1/2, *m_F_ = ±F*. The sensor axis passes through the centers of cells C1 and C2 in the direction of light propagation—along the *x*-axis in Figure 1. When the sensor is rotated around its axis, the parallelism of vectors E and B can be ensured by choosing the direction of the HWP axis. This will make it possible to rotate the sensor around its axis by 360° without degrading its parameters, which should be considered an additional advantage of the proposed scheme.

The block of the optical scheme, which requires preliminary calculation, is enclosed in a dotted rectangle in Figure 1. Two problems were solved: (1) conversion of the input linearly polarized light with modulated azimuth into the light with the required polarization parameters, and (2) preservation of the polarization parameters of the light when the beam is split into two beams necessary for pumping and interrogating two cells. The ultimate goal of optimization was to ensure identical characteristics of the beams in the two cells in all phases of modulation.

The second task turned out to be non-trivial since any beam-splitting mirror, as well as any interference beam splitter, either changes the ratio of the intensities of the *s* and *p* radiation components or introduces a significant phase delay between them. Of the possible solutions, we chose the most compact one: rotating the beam polarization azimuth in front of the beam-splitting mirror and introducing a neutral filter into one of the channels. The rotation is carried out by rotating the linear polarizer frame; after passing through the beam-splitting unit, it has to be compensated by additional HWPs.

To calculate the optical scheme, we used the formalism of Mueller matrices [41]. The Stokes vector of radiation that has passed through a number of optical elements is described by successive multiplication by matrices corresponding to these elements. Thus, the Stokes vectors in two cells can be described by the expressions:

(1) $$\begin{array}{l} S_{1}=M_{HWP}M_{NTM}M_{STM-T}M_{RLP}M_{QWP}S_{0};\\ S_{2}=M_{HWP}M_{NF}M_{STM-R}M_{RLP}M_{QWP}S_{0}, \end{array}$$

where *S*_0_ is the Stokes vector of the input beam, *M_NTM_* is the non-transparent mirror matrix, *M_STM-T_* is the semitransparent mirror matrix for the transmitted beam, *M_STM-R_* is the semitransparent mirror matrix for the reflected beam, *M_NF_* is the neutral density filter matrix, *M_RLP_* is the variable linear polarizer array, *M_QWP_* is quarter-wave plate matrix, *M_HWP_*—half-wave plate matrix. A stack of *N* plane-parallel glass plates located at the Brewster angle (*M_RLP_* = *M_G_^N^*, one glass is described by the *M_G_* matrix [42]) was used as a regulated linear polarizer.

The Mueller matrices used in our calculations are given in Appendix A. During the optimization, the following parameters varied: α, the RLR rotation angle, and *T_NF_*, which is the density of the neutral filter.

**Figure 2:**
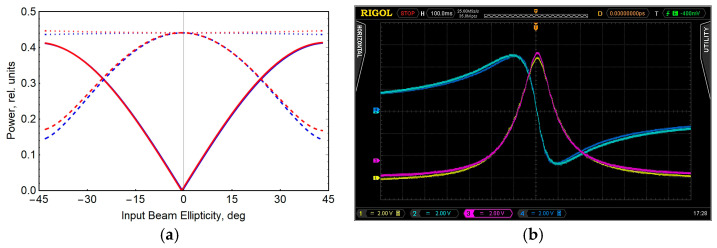
(**a**) Example of calculation results: red lines are the light intensity at the input to cell C1, and blue lines are the light intensity at the input to cell C2. Solid lines are the circular component; dashed lines are the linear component; dotted lines are the total intensity. (**b**) Oscillograms of the magnetic resonance signals in cells C1 and C2 after synchronous detection (one component and MR signal module are shown).

Figure 2a shows the calculation result for the optical elements used in our experiment. The reflection and transmission coefficients of the beam-splitting mirror for the *s* component are *R_s_* = 0.72 and *T_s_* = 0.28, respectively, and for the *p* component, *R_p_* = 0.37 and *T_p_* = 0.63. The reflection coefficients for an opaque silver mirror for the s and p components are *R_s_* = 0.997 and *R_p_* = 0.976, respectively. Equalization of radiation parameters in two cells is achieved at α = 46° and *T_NF_* = 0.82.

Oscillograms of MR signals in two cells after synchronous detection (one component and MR signal module) are also shown (Figure 2b). As Figure 2b illustrates, the amplitudes and widths of the resonances in the cells are approximately the same, and there is no frequency shift between the resonances, which indicates a good balance of the light parameters in the two cells.

## 3. Results

**Figure 3:**
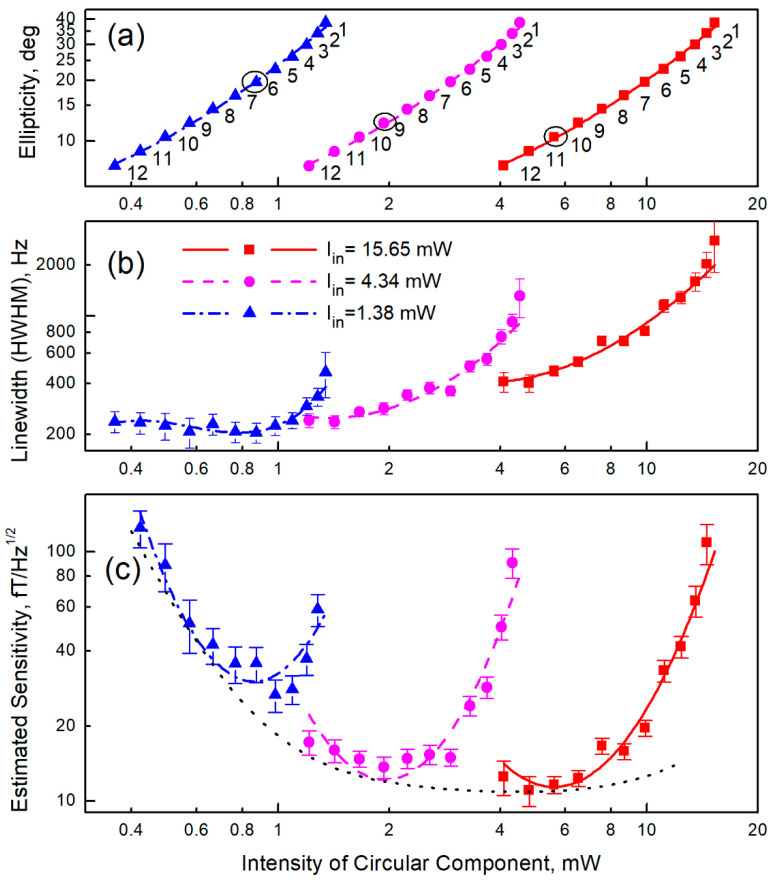
Dependence of the parameters of magnetic resonance when pumped with light with modulated ellipticity on the light intensity at the input of the cell: (**a**) ellipticity for different numbers (indicated by numbers in the graph field) of glass plates in a linear polarizer; the black circles indicate the optimal ellipticity values for this series, (**b**) magnetic resonance half-width, (**c**) estimation of the ultimate (limited by calculated shot noise) sensitivity. Connecting lines are guides to the eye.

Differences in the radiation characteristics in the proposed scheme from those required in [25] are reduced to the fact that the ellipticity modulation is carried out to a rectangular law (Figure 1) instead of a sinusoidal one. Thus, both the circular and linear components are characterized by constant intensities, and the phases of MR signal detection are not separated in time from the pump phases. The influence of the modulation shape in the standard two-beam Bell–Bloom scheme was studied in [40], and it was shown that although rectangular modulation leads to a slight broadening of the MR signal, it nevertheless allows values close to the ultimate sensitivity to be reached; however, the assumption that this is also true for the single-beam scheme requires proof. Therefore, we simulated the pumping conditions during the light transmission by the method described above and studied the MR parameters. The measurement results are shown in Figure 3. As in [25], we estimated the ultimate short-term sensitivity by calculating the ratio of the measured resonance amplitude to its measured width and to the calculated spectral density of the photocurrent shot noise.

In accordance with the results presented in Figure 3, the required value of ellipticity (Figure 3a) was chosen according to the criterion of maximum sensitivity (Figure 3c), based on the available intensity of laser light and the value of losses on the elements of the optical scheme. As a consequence, the parameters of the linear polarizer (the number of glass plates in a stack) and the light intensity in each cell were determined (see Section 4).

**Figure 4:**
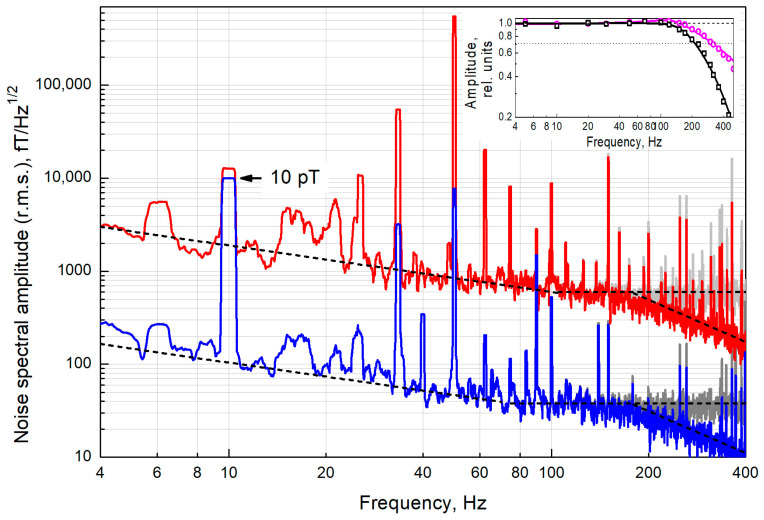
Noise spectrum of the magnetic resonance signal in cell C2 (red line), and the difference signal of magnetic resonances in cells C1 and C2 (blue line)—moving r.m.s. average in 1 Hz band. Gray lines are the spectra corrected for the frequency response of the sensor. The peak at a frequency of 10 Hz (marked with an arrow) is a calibration signal with an amplitude of 10 pT r.m.s. The peak at a frequency of 50 Hz is the interference from the main currents. The dashed lines are the noise floors of the signal in cell C2 and of the difference signal, respectively. Inset: the magenta line is the sensor’s frequency response (cutoff frequency *f*_0_ = 315 Hz), the black line is the sensor’s frequency response, taking into account the time constant of the SR830 synchronous detector (τ = 0.3 ms, 18 dB/octave).

Next, we measured the gradiometric sensitivity of the proposed scheme when pumped with linearly polarized radiation with modulated azimuth. To do this, a magnetic coil was mounted on the frontal plane of the thermostat. The field generated by the coil in each of the cells was measured by the displacement of the magnetic resonance line. Based on the response to the same field, the frequency band of the sensor was determined: *f*_0_ *=* Γ/(*2π*) ≈ 315 Hz. Further, in the experiment, the response speed was additionally limited by the time constant of the synchronous detector (τ = 0.3 ms, 18 dB/octave). The measurement results are shown in Figure 4.

## 4. Discussion

Let us try to evaluate how the proposed changes in the sensor design affect its ultimate characteristics, the most significant of which are the achievable sensitivity and bandwidth. For this, we compare the MR parameters obtained in this work with the parameters obtained in [25]. According to the evaluation given in [25], the shot-noise-limited sensitivity reached 8.8 fT/√Hz at a bandwidth (determined by the MR width) of the order of Γ/(2π) ≈ 580 Hz, whereas, according to Figure 3, the shot-noise-limited sensitivity reaches (11.0 ± 0.7) fT/√Hz at a bandwidth of Γ/(2π) ≈ 430 Hz. These results show that the proposed scheme can be used in MEG complexes without noticeable deterioration in their parameters.

**Figure A1:**
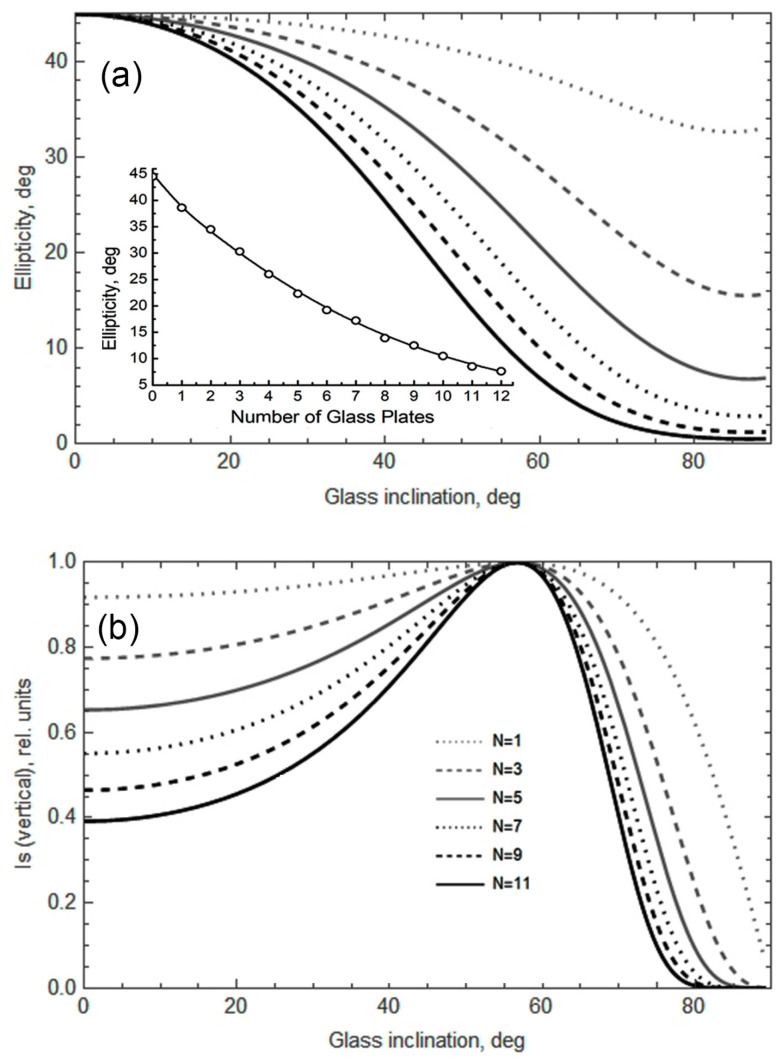
Characteristics of the output radiation of a regulated linear polarizer consisting of *N* thin glass plates-calculation by Formulas (A5)–(A9); the input light is circularly polarized. (**a**) Dependence of the ellipticity of the output light on the angle of inclination of the glass plates; Inset: ellipticity of the output light as a function of the number *N* of glass plates set at the Brewster angle. The line is the calculation; the circles are the experiment. (**b**) Dependence of the transmission of the *s*-component of radiation on the angle of inclination of the glass plates.

The difference in sensitivity is explained, in particular, by the additional light loss in the linear polarizer. The optimal value of ellipticity lies in the range of 10–20° (Figure 3a,c), which is fully consistent with the data [25]. With the intensity available to us in one cell (roughly corresponding to the magenta series in Figure 3), the ellipticity of (11 ± 1)° is optimal. This means that (47.9 ± 0.3)% of the total intensity is lost in an ideal adjustable linear polarizer. As a polarizer, we used a stack of conventional microscope coverslips. Due to the imperfection of the surfaces and the spread of their installation angles, the loss on a stack of 9–10 glass plates providing the corresponding ellipticity (see Figure A1) amounted to (66.7 ± 0.9)%. Under the conditions of limited laser power (15.65 mW at the EOM output); this loss forced us to reduce the working cell temperature to ~80 °C compared to 90 °C in [25].

It should be noted that the data in Figure 3 were obtained without using a beam splitter, i.e., all the light intensity was fed into one cell. When we operate with two cells (Figure 4), the power available in our experiment in each channel is ~40% of the maximum (see Figure 2a),—i.e., about 2.1 mW per cell. As a result, the ultimate shot-noise-limited sensitivity deteriorates to the value of (15.1 ± 0.7) fT/√Hz, and a MR half-width Γ is reduced to 2π∙350 Hz.

The MR width, in addition to the bandwidth, also determines the permissible field inhomogeneity, that is, the maximum difference in magnetic fields at the points of location of individual sensors. Thus, at half-width Γ = 2π∙350 Hz, the maximum allowable deviation of the field from the array-average value for a sensor based on cesium atoms will be approximately *k∙*Γ/*γ_Cs_* ≈ 50 nT (here, *γ_Cs_* ≈ 2π∙3.5 Hz/nT is the gyromagnetic ratio Cs, *k* ≈ 0.5 is the width of the conditionally linear section on the dispersion contour of the MR, referred to Γ). An array radius of 0.1 m corresponds to an allowable gradient of 1 μT/m. 

If we exclude from the spectra in Figure 4 the zones of technical interference and technical noise that dominates at low frequencies (up to 80 Hz), the gradient noise lies in the range of 30–60 fT/√Hz. In terms of one channel of the sensor, this is 20–40 fT/√Hz, and approximately corresponds to the sensitivity limit estimate given earlier in this section. In addition to photon shot noise, the contribution to the white noise recorded at frequencies above 80 Hz can come from both technical factors (white thermal Johnson noise) and fundamental ones (atomic projection noise). The atomic projection noise amplitude with the optimal parameter configuration is comparable to the shot noise amplitude.

According to [43], in our cylindrical shield, in which the radius of the inner shell made of steel is *a* = 17 cm, the thermal noise amplitude should be ~23 fT/√Hz. The noise suppression coefficient in the gradiometric scheme in this shield should be about 1.19∙(*d/a*), where *d* is the distance between the cells. In our experiment, *d* = 1.0 cm, which corresponds to noise suppression by a factor of 20, down to 1.1 fT/√Hz. The value of the thermal noise component proportional to *f*^−1/2^ should also not exceed units of fT/√Hz at a frequency of 1 Hz [43]. Thus, the thermal noise of the shield should not make a significant contribution to our measurements.

The external field’s suppression level in the gradiometric scheme can be estimated from the suppression of pickup at a frequency of 50 Hz: it is suppressed approximately 70-fold. We can take the residual pickup level (~1.4%) as an upper bound for the unbalance of the gradiometer parameters.

At the same time, both the *f*^−1/2^ noise, which dominates at frequencies up to 80 Hz, and white noise, which dominates at frequencies above 80 Hz, are suppressed much less, approximately by a factor of 16√2 ≈ 23 (taking into account that two channels contribute to the noise of the difference signal). This can be explained by laser radiation noise, both intrinsic and acoustic, during the transmission of radiation through the air over a distance of ~2 m. Thus, to further improve the scheme, it is necessary, first, to increase the power of laser radiation (taking into account the inevitable losses during input into the SM-PM fiber) and, second, to actively stabilize its parameters.

## 5. Conclusions

We have shown that the earlier proposed scheme can be modified to exclude the transmission of elliptically polarized radiation from the pump source to the sensor—which makes it possible to use optical fiber for radiation transmission. This eliminates the last fundamental obstacle to constructing a magnetoencephalographic system of a non-zero field based on single-beam optical sensors. A magnetometer-gradientometer based on this principle has demonstrated a limiting sensitivity (estimated from the ratio of signal to linewidth and photon shot noise) at the level of (11.0 ± 0.7) fT/√Hz at the optimum optical pump intensity and 15–18 fT/√Hz at the distribution of pump radiation on two sensor channels. Direct measurement of the gradiometric sensitivity of the proposed scheme showed that the sensitivity of one sensor channel in the range of 80–200 Hz reaches 20 fT/√Hz. Further improvement in sensitivity can be achieved by using a more powerful laser pump source with a fiber output and active methods for suppressing laser radiation noise.

## Appendix A

The Muller matrix [41] for neutral filter:

(A1) $$M_{NF}=T_{NF}\left(\begin{array}{cccc} 1 & 0 & 0 & 0\\ 0 & 1 & 0 & 0\\ 0 & 0 & 1 & 0\\ 0 & 0 & 0 & 1 \end{array}\right),$$

where *T_NF_* is the transmittance of the neutral filter.

Muller matrix for the phase plate (the expression is used to calculate the matrices *M_QWP_*, *M_HWP_*):

(A2) $$M_{WP}=\left(\begin{array}{cccc} 1 & 0 & 0 & 0\\ 0 & Cos^{2}\left(2\Theta \right)+Sin^{2}\left(2\Theta \right)Cos\left(\delta \right) & Cos\left(2\Theta \right)Sin\left(2\Theta \right)\left[1-Cos\left(\delta \right)\right] & Sin\left(2\Theta \right)Sin\left(\delta \right)\\ 0 & Cos\left(2\Theta \right)Sin\left(2\Theta \right)\left[1-Cos\left(\delta \right)\right] & Cos^{2}\left(2\Theta \right)Cos\left(\delta \right)+Sin^{2}\left(2\Theta \right) & -Cos\left(2\Theta \right)Sin\left(\delta \right)\\ 0 & -Sin\left(2\Theta \right)Sin\left(\delta \right) & Cos\left(2\Theta \right)Sin\left(\delta \right) & Cos\left(\delta \right) \end{array}\right),$$

where Θ is the angle of rotation of the main axis of the plate, δ is the phase delay angle (equal to π/2 for QWP, and π for HWP).

Muller matrix for a mirror (the expression is used to calculate the matrices *M_NTM_*, *M_STM-T_*, *M_STM-R_*)):

(A3) $$M_{M}=\left(\begin{array}{cccc} \frac{R_{p}+R_{s}}{2} & \frac{R_{p}-R_{s}}{2} & 0 & 0\\ \frac{R_{p}-R_{s}}{2} & \frac{R_{p}+R_{s}}{2} & 0 & 0\\ 0 & 0 & -\sqrt{R_{p}R_{s}}Cos\left(\delta \right) & -\sqrt{R_{p}R_{s}}Sin\left(\delta \right)\\ 0 & 0 & \sqrt{R_{p}R_{s}}Sin\left(\delta \right) & -\sqrt{R_{p}R_{s}}Cos\left(\delta \right) \end{array}\right),$$

where *R_p_* and *R_s_* are the reflection coefficients for p and s polarizations, respectively, and δ is the phase delay angle. When calculating the transmission through a semitransparent mirror, the reflection coefficients are replaced by the transmission coefficients *T_p_* = 1 − *R_p_*, *T_s_* = 1 − *R_s_*, and the sign of δ is inverted.

In general, the normal mirror plane is not parallel to the beam. If the axis of rotation of the mirror does not coincide with the axis of the coordinate system, the Muller matrix of the mirror is transformed using the matrix *M_R_*, which describes the rotation of the polarization plane through the angle Θ:

(A4) $$M_{M’}=M_{R}(\Theta )M_{M}M_{R}(-\Theta )=M_{R}M_{M}M_{R}{}^{-1}$$

where

(A5) $$M_{R}=\left(\begin{array}{cccc} 1 & 0 & 0 & 0\\ 0 & Cos\left(2\Theta \right) & Sin\left(2\Theta \right) & 0\\ 0 & -Sin\left(2\Theta \right) & Cos\left(2\Theta \right) & 0\\ 0 & 0 & 0 & 1 \end{array}\right),$$

Expression (A5) is also applicable to the calculation of the matrix *M_SF_*(*n*_1_,*n*_2_,*φ*), which describes reflection and refraction when light is incident at an angle φ on the boundary of two media with refractive indices *n*_1_ and *n*_2_, and the coefficients *R_p_*(*n*_1_,*n*_2_,*φ*), *R_s_(n*_1_,*n*_2_,*φ*), *T_p_*(*n*_1_,*n*_2_,*φ*), *T_s_*(*n*_1_,*n*_2_,*φ*) are described by Fresnel equations [42].

The matrix *M_GL_*(*n*_1_,*n*_2_,*φ*) describes the through passage of a beam through two surfaces of a plane-parallel glass plate:

(A6) $$M_{GL0}\left(n_{1},n_{2},\varphi \right)=M_{SF}\left(n_{2},n_{1},\psi \right)M_{SF}\left(n_{1},n_{2},\varphi \right).$$

where *ψ* is the direction of the refracted beam inside the plate: sin(*ψ*) *= (n*_1_/*n*_2_)sin(*φ*).

If the beam displacement is large compared to the beam diameter, subsequent re-reflections can be neglected and vice versa. If the plate thickness is small compared to the beam diameter, the beam shift due to re-reflections can be neglected. Then, the expression for the transmission of a plane-parallel glass plate *M_GL_*(*n*_1_,*n*_2_,*φ*) must be constructed by summing an infinite series describing multiple reflections from two surfaces:

(A7) $$M_{GL}\left(n_{1},n_{2},\varphi \right)=M_{SF}\left(n_{2},n_{1},\psi \right)\sum_{i=0}^{\infty }{\left[1-M_{SF}\left(n_{2},n_{1},\psi \right)\right]}^{2i}M_{SF}\left(n_{1},n_{2},\varphi \right),$$

Consequently,

(A8) $$M_{GL}\left(n_{1},n_{2},\varphi \right)={\left[2-M_{SF}\left(n_{2},n_{1},\psi \right)\right]}^{-1}M_{SF}\left(n_{1},n_{2},\varphi \right).$$

Accordingly, a stack of *N* plates located at an angle *φ* to the beam direction is described by the matrix:

(A9) $$M_{RLP}\left(n_{1},n_{2},\varphi ,\Theta \right)=M_{R}\left(\Theta \right){\left(M_{GL}\left(n_{1},n_{2},\varphi \right)\right)}^{N}M_{R}\left(-\Theta \right).$$

Since the RLP must provide partial suppression of one linear component with the maximum transmission of another, the angle *φ* should be chosen equal to the Brewster angle: *φ = φ_Br_ =* arctg(*n*_2_/*n*_1_). The calculated linear polarizer parameters are shown in Figure A1.
